# Design, Expression and Purification of *Strongyloides stercoralis* IgG4 Immunoreactive Protein (NIE) in *Escherichia coli*

**DOI:** 10.18502/ijpa.v15i3.4198

**Published:** 2020

**Authors:** Katayoun DASTAN, Mehdi ASSMAR, Nour AMIRMOZAFARI, Fariborz Mansour GHANAEI, Mirsasan MIRPOUR

**Affiliations:** 1. Department of Microbiology, Faculty of Basic Sciences, Lahijan Branch, Islamic Azad University, Lahijan, Iran; 2. Department of Parasitology, Pasteur Institute of Iran, Tehran, Iran; 3. Department of Microbiology, School of Medicine, Iran University of Medical Sciences, Tehran, Iran; 4. Division of Gastroenterology, Faculty of Medicine, Gilan University of Medical Sciences, Gilan, Iran; 5. Gastrointestinal and Liver Disease Research Center, Razi Hospital, Rasht, Iran

**Keywords:** *Strongyloides stercoralis*, Immunodiagnostic, Protein expression

## Abstract

**Background:**

Strongyloidiasis is a public health concern in northern regions of Iran, caused by *Strongyloides stercoralis*. Auto-infection cycle can be resulted in high parasitic load, especially in immunocompromised hosts. Because of low sensitivity of stool culture and stool-based microscopy techniques, detection of antibodies in patient’s sera can be an alternative diagnostic technique for detection of the nematode. In the present study, as the first step of the development of an ELISA kit for the detection of antibodies against the nematode, IgG4 immunoreactive protein (NIE) was expressed in *Escherichia coli* expression system, purified and verified.

**Methods:**

The *NIE* gene sequence was retrieved from the GenBank. This sequence was codon-optimized for the expression in *E. coli* BL21 (DE3). The sequence was inserted into the expression vector pET-30b (+). The recombinant vector was then transferred into competent *E. coli* BL21 (DE3). Transformed colonies were selected and verified by colony PCR. *NIE* gene expression was induced with IPTG induction. The protein production was evaluated by SDS-PAGE and verified using Western blotting.

**Results:**

The codon-optimized *NIE* gene had required parameters for expression in *E. coli*. NIE protein was proved and verified by SDS-PAGE and Western blotting.

**Conclusion:**

*NIE* recombinant protein was successfully expressed in *E. coli* expression system in appropriate amounts. The recombinant protein can be used for developing ELISA kit in diagnosis of *S. stercoralis.*

## Introduction

Strongyloidiasis is a tropical neglected disease, caused by a soil-transmitted helminth, *Strongyloides stercoralis*. The nematode infects up to 100 million people worldwide annually (1). The nematode has been reported from different areas in Iran and there are many studies evaluated the prevalence of the nematode in different rural and urban areas of the country (2–5). However, there has not been a comprehensive study for evaluating the prevalence of nematode in Iran.

Because of the special life cycle of *S stercolaris,* in areas where the nematode is endemic, chronic infections may be acquired and last for decades through the autoinfection cycle, without any detectable symptoms (6). Therefore, it is of great importance to have specific and sensitive tests for the diagnosis of the nematode, especially in immunocompromised and elderly people, to prevent further distribution as well as more mortality rate due to the infection (7, 8). Because of the lack of production of characteristic ova in the gastrointestinal tract, it is necessary to directly observe the larva of *S stercolaris* in stool culture. However, since the larval concentration of the nematode is generally low, the sensitivity of stool-based methods for the detection of the nematode is as low as 30%–50% (9), which is a big challenge, particularly in patient with chronic form. Indeed, due to the infectiveness of the contact to this nematode, laboratory personnel are at great risk for this infection (10). Considering the difficulties of stool-based methods, surrogate detection of the nematode using serological methods is a very important issue. There are many studies used this strategy for the detection of *S stercolaris*, most of them relied on the detection of anti-NIE IgG (11–13). However, different antigens have been also used for this purpose (11, 14).

NIE antigen, which firstly introduced in 2002 (15), can stimulate the release of histamine from basophils and has been used in different studies for immunodiagnosis of *S. stercoralis* (16, 17). Antibody detection assays that exploited this antigen for the detection have had proper sensitivities (84%–98%) and specificities (95%–100%) (12).

Because of the high sensitivity and specificity of this antigen, in the present study, *NIE* protein was expressed, purified and verified, as the first step of the development of an ELISA kit for the detection of antibodies against the nematode.

## Materials and Methods

### Chemicals, Enzymes and Media

Luria-Bertani broth and agar were purchased from Difco Laboratories (USA). Chemical re-agents were mainly purchased from Merck (Germany). Glacial acetic acid and ethanol (96%) were prepared from Mojallali co. (Iran). Kanamycin, ampicillin and anti-His tag antibody were purchased from Roche (Germany). Isopropyl β-D-1-thiogalactopyranoside (IPTG) was prepared from SinaClon (Iran). PCR master mix and 1 kb DNA Ladder were purchased from GoldBio(China).

### Adoption and codon optimization of NIE gene sequence

The NIE sequence was adopted from Gen-Bank with the accession number of AAB97359. Codon optimization of the gene was performed using OPTIMIZER software (18) and GenScript’s patented OptimumGene^TM^ was exploited for further analysis (https://www.genscript.com/tools/). Determination of the mRNA secondary structure using mfold server (http://unafold.rna.albany.edu/) was conducted to analyze the stability of the mRNA as well as investigate the accessibility of the ribosome binding site (RBS). After codon-optimization, the sequence was chemically synthesized in pET30a (+) by Bioneer Company (South Korea).

### Transformation of the bacterial cells

The recombinant construct was transferred into *E. coli* DH5α competent cells via heat shock method (19). Competent *E. coli* cells were prepared by CaCl_2_ method (20). To confirm the transformation of the bacteria, plasmid extraction was performed by plasmid extraction kit (Bioneer, Korea) according to the manufacturer’s instructions. PCR reaction was exploited to confirm the transformation of the bacteria.

### NIE protein expression

*E. coli* BL21 (DE3) was used for the expression of the recombinant protein. After the transformation of bacteria with the recombinant vector, NIE protein was expressed. For this aim, the protocol described by Hajizade et al., was used (21). Shortly, the transformed bacteria were inoculated into the LB (Luria-Bertani) broth medium. Once the optical density of the culture medium at 600 nm reached 0.6, IPTG (with the final concentration of 1mM) was added to the media and the expression was performed for 4 h. Then, the expression of the protein was investigated on a 12% SDS-PAGE. For this purpose, 2 ml of the media was centrifuged at 10000 g for 2 min. The supernatant was discarded and 300 μl of lysis buffer (10 mM tris-HCl, 100mM NaH_2_PO_4_, 8M urea, pH=8) was added to the pellet. The mixture was shaken in a shaker incubator for 1 h at 37 °C. The mixture was centrifuged at 20000 g for 30 min at 4 °C. 20 μl of the supernatant was loaded onto a 12% SDS-PAGE to analyze the protein expression.

### Confirmation of the protein expression by Western blotting

Western blot analysis was used to confirm the expression of NIE recombinant protein. For this purpose, the method described by Sayadmanesh et al., was exploited (22). Briefly, following the electrophoresis of NIE protein on a SDS-PAGE, it was transferred on a nitrocellulose membrane. The membrane was blocked through an overnight incubation of the membrane in 5% w/v of Skimmed milk in PBST a 4 °C. Following the wash with PBST, the membrane was incubated in PBST containing mouse anti-His antibody (Abcam, USA, 1:5000). Then, the membrane was washed and incubated in PBST containing HRP-conjugated anti-mouse IgG and finally, the expression of the NIE protein was investigated by addition of substrate solution (DAB, Tris 50 mM, pH 8 and H_2_O_2_).

### NIE protein purification by affinity chromatography

Since the expressed NIE protein had a histidine-tag, a nickel chromatography column (Sigma, Germany) was used to purify the protein. The purification was performed under the non-denaturing condition (23). For this, 100 ml culture of the transformed bacteria was prepared and protein expression was induced by the addition of 1mM IPTG. Then, the cells were harvested and 10 ml of lysis buffer (10 mM HCl, 100 mM NaH_2_PO_4_, 10 mM imidazole pH 7.4) was added to the cells. After, 1 h shaking in an incubator at 37 °C, the lysate was centrifuged (at 20000 g for 30 min). The supernatant was passed through a nickel column and buffers C, D and E were applied to the column respectively. The compositions of buffers C, D and E were as the same as lysis buffer, unless their pH was 6.4, 5.9 and 4.5, respectively. After applying each buffer, the flow-through was collected and was analyzed by a 12% SDS-PAGE.

Determination of the protein concentration was performed using Bradford method (24).

## Results

### Bioinformatics analyses

**Fig. 1:**
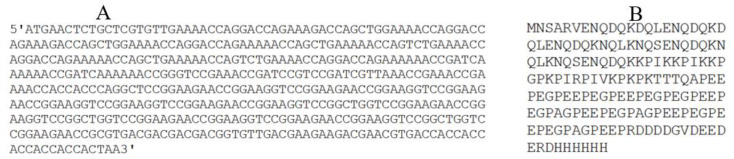
Sequence of the codon-optimized *NIE* gene (A) along with the amino acid sequence of the NIE protein (B)

**Fig. 2:**
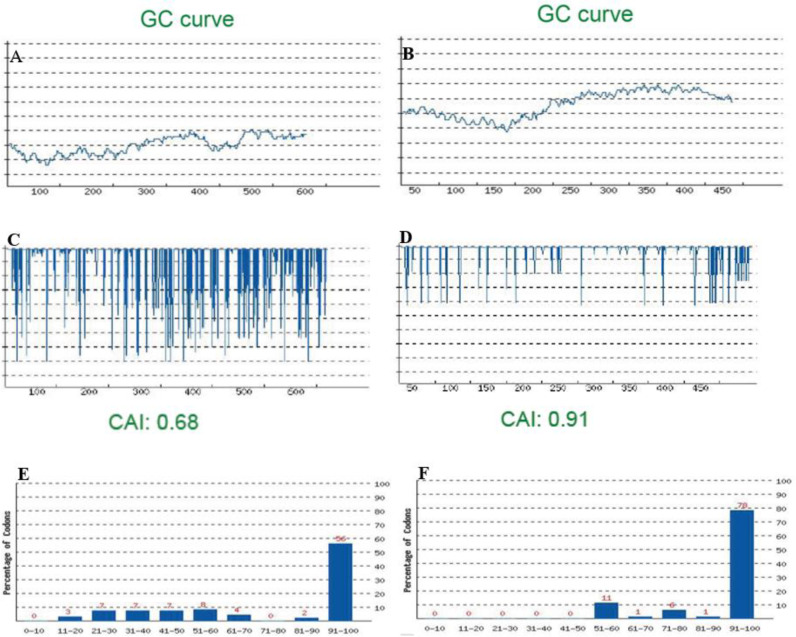
Bioinformatic analysis of the *NIE* gene before and after the codon-optimization. All unfavorable characteristics of the original sequence are addressed following the codon optimization. A and B: The GC content of the *NIE* gene before and after the codon-optimization, respectively. C and D: Codon adaptation index of the *NIE* gene before and after the codon-optimization; E and F: CFD of the two sequences

The codon-optimized sequence of the *NIE* gene has been presented in Fig. 1 and GC content, codon frequency distribution (CFD) and codon adaptation index (CAI) are illustrated in Fig. 2. GC content of the sequence was 28.92% and 55.25% before and after the optimization, respectively (Fig. 2). Analysis of CFD showed that there was no rare codon in the optimized sequence (Fig. 2). Codon Adaptation Index (CAI) of the sequence was 0.68 and 0.91, respectively before and after the optimization, which demonstrates that the sequence can be efficiently expressed in *E. coli* BL21 (Fig. 2). Analysis by OPTIMISER also revealed that the effective number of codons (ENC) has changed from 33 to 20 after the codon-optimization. Secondary structure of the resulted mRNA by using mfold server showed that the mRNA is stable inside the cell and its ribosome binding site (RBS) is accessible for ribosome entry and translation initiation. According to ProtParam program, the sequence is stable in *E. coli* and can be expressed in these cells.

### Confirmation of the E. coli transformation

**Fig. 3:**
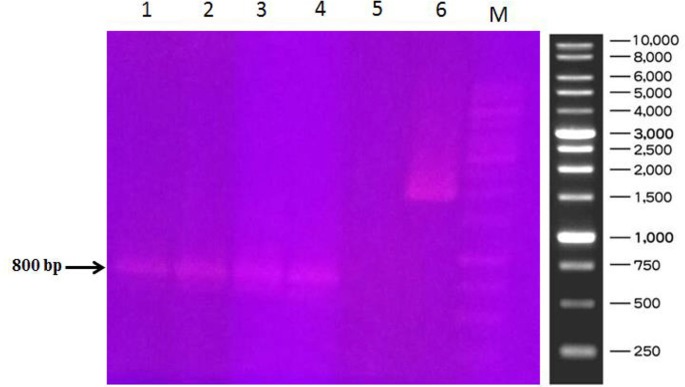
Colony PCR of the grown colonies on LB agar supplemented with Kanamycin. Lanes 1–4: Four different colonies grown on the LB agar; Lane 5: Negative control. Lane 6: Positive control; M: 1 kb DNA Ladder. In negative control, all materials of the PCR reaction were as the same as the test samples but the template DNA, which was the genomic DNA of *E. coli* BL21 (DE3) strain. In positive control, another DNA sequence (with a size of about 2 kb) with its primers was used

The plasmid carrying the *NIE* gene was transferred into the competent *E. coli* cells. The transformation of both *E. coli* DH5α and *E. coli* BL21 cells was confirmed by PCR reaction using pET30b (+) universal primers (Fig. 3). Since the PCR has been performed using T7 promoter and terminator universal primers, a 90 bp and a 174 bp fragment are respectively added to the 5′ and 3′ ends of the gene, so the amplified fragment has a size of around 800 bp. Negative and positive controls were applied to check the PCR reaction. In negative control, all materials of the PCR reaction were as the same as the test samples but the template DNA, which was the genomic DNA of untransformed *E. coli* BL21 (DE3) strain. The positive control was added to check if the materials of the PCR reaction, including PCR buffer, DNA polymerase, dNTPs and MgCl2 buffer work properly, so in positive control another DNA sequence (with a size of about 2 kb) with its own specific primers were exploited. No amplified PCR product is present in negative control but in the positive control, the desired DNA sequence has been amplified successfully (Fig. 3).

### NIE protein expression and Western blotting

**Fig. 4:**
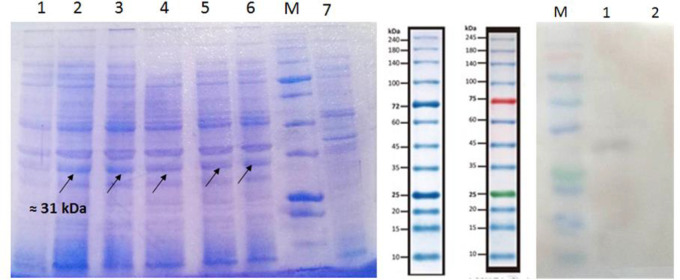
A. Induction of NIE protein Expression by addition of IPTG. Lanes 1 and 2: Sample A, 4 and 14 h of induction, respectively; Lanes 3 and 4: Sample B, 4 and 14 h of induction; Lanes 5 and 6: Sample C, 4 and 14 h of induction; M: Protein size marker; Lane 7: Un-induced sample. B. Western blot analysis using anti-His tag antibody. Lanes 1 and 2: Induced and un-induced samples, respectively

SDS-PAGE analysis showed the presence of a 31 kDa recombinant protein in all three induced samples (Fig. 4A). No significant difference was observed among the three colonies or in the applied periods (4 and 14 h). One sample was selected for investigating the using Western blot analysis. Western blotting was performed using anti-His tag antibody to verify the accuracy of the expressed protein and, it confirmed the expression of the recombinant NIE protein (Fig. 4B).

### Purification of NIE protein

**Fig. 5:**
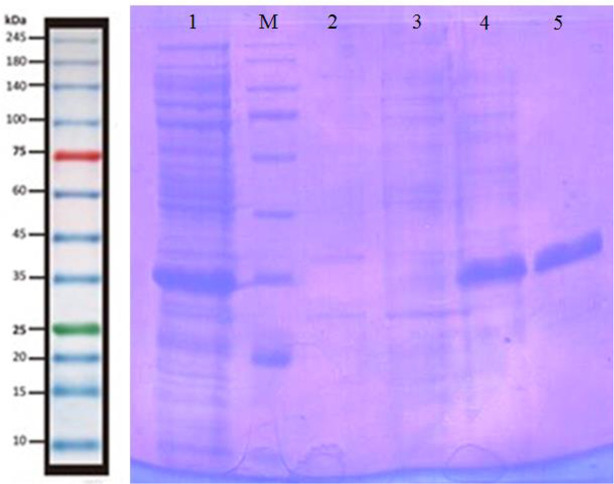
Purification of NIE protein using a nickel column. Lane 1: Protein solution before applying to the column. Lane 2: Flowthrough of the column following applying the protein solution to the column. Lanes 3–5: Flowthrough of the column following the addition of 40 mM (lane 3), 100 mM (lane 4) and 250 mM imidazole buffer (lane 5)

Results of solubility assessment showed that the protein is expressed as a soluble protein (data not shown), so the protein was purified using Ni-NTA under non-denaturing conditions (Fig. 5). The purified protein was obtained following the addition of 250 mM imidazole buffer.

## Discussion

A sensitive, robust, and rapid detection method for the diagnosis of *S stercoralis* can reduce the nematode’s distribution and, as a result, morbidity and mortality due to this parasite. In the present study, as the first step for the development of a detection kit for the diagnosis of *S stercoralis*, NIE protein was expressed in *E. coli* expression system, confirmed by Western blotting and purified by affinity chromatography using a nickel column.

NIE was firstly introduced in 2002 as a promising candidate antigen for immunodetection of *S. stercoralis* (15). Since then many studies have exploited this protein for this aim. A luciferase immunoprecipitation system assay was investigated based on NIE protein to diagnose *S. stercoralis* infection and reached the specificity of 100% (11, 16). NIE protein was used to develop a rapid and sensitive immunodetection method for strongyloidiasis using diffraction-based optical biosensor technology. The authors could diagnose the disease in less than 30 min with a sensitivity and specificity of 98% and 100%, respectively (17). These and many other studies have demonstrated the efficiency of NIE protein in immunodiagnosis of *S. stercoralis* infection (13, 16).

Since the protein is originally expressed in a parasite, there was a low possibility of the expression of the NIE-encoding gene in *E. coli* expression system. Therefore, the gene was codon-optimized according to *E. coli* codon usage. By codon optimization, all main affecting parameters on the successful expression of the gene, including CAI, ENC and GC% were in the optimal situation. CAI of the optimized gene was changed from 0.64 to 0.91. Generally, the lower the CAI number of a gene, the poorer expression of that gene (25). In the original sequence, ENC, which reflects the presence of the different codon types in a sequence, was 33, while after the codon-optimization, with the deletion of the rare codons, it reached to 20. Since the presence of the rare codons lowers the rate of translation elongation (due to the lower supply of tRNA) (26, 27), the expression efficiency will be reduced. Here, the issue was addressed by codon-optimization of the sequence. Indeed, prediction of the mRNA secondary structure demonstrated the stability of the molecule (ΔG: −170 kCal/mol) as well as the accessibility of the RBS which causes mRNA to be efficiently recognized by ribosomes (28).

In this study, pET30b (+) expression vector was exploited for the expression of the desired protein. Since in the pET series vector the MCS (multiple cloning site, where gene or DNA sequence of interest will be inserted within it) is located downstream of the T7 promoter, genes cloned in pET expression vectors will be efficiently transcribed in DE3 strains of *E. coli* cells, in whose T7 RNA polymerase has been incorporated into the bacterial genome. Since *NIE* gene has been inserted between NdeI and BamHI restriction sites, the N-terminal histidine-tag will not be appeared in the expressed protein, so a CACCACCACCACCACCAC sequence, which encodes for a 6His-tag, was added to the 3′-end of the gene for easy purification of the expressed protein by using a nickel column. Using the mentioned strategies, including codon optimization, appropriate vector and bacterial strain, purification by affinity column, the efficiency of the protein expression and purification was desirable so that the protein yield was determined as 15 mg/L culture medium.

Theoretical molecular weight of the recombinant NIE protein, calculated by ProtParam server, tends to be about 18 kDa. However, when it was electrophoresed on a 12% SDS-PAGE, the protein has appeared as about a 30 kDa protein. Western blotting, as well as purification of the protein by nickel column, confirmed the accuracy of the expressed protein. The difference between the theoretical and apparent molecular masses of NIE protein can be explained by the high GRAVY (grand average of hydrophobicity) index of the protein (−2.165, as calculated by http://www.bioinformatics.org/) and its low isoelectric point (4.45, as calculated by Prot-Param server). Although SDS-PAGE is frequently used for molecular size determination of a protein, however, since SDS binds to hydrophobic amino acids more efficiently than charged ones, especially negatively charged amino acids and, as a result, the theoretical mobility is different from the apparent mobility (29). In the case of NIE protein, due to the high frequency of the negatively charged amino acids, i.e. glutamate and aspartate, SDS binds to the protein much poorer and the protein migrates more slowly than it is expected.

## Conclusion

NIE protein, an immunodiagnostic protein of *S. stercoralis* can be used as a target for the development of an ELISA kit for immune-detection of the nematode. The protein was successfully expressed and purified and might be used for further future studies.
